# Coilin association with Box C/D scaRNA suggests a direct role for the Cajal body marker protein in scaRNP biogenesis

**DOI:** 10.1242/bio.20147443

**Published:** 2014-03-21

**Authors:** Isioma I. Enwerem, Venkatramreddy Velma, Hanna J. Broome, Marija Kuna, Rowshan A. Begum, Michael D. Hebert

**Affiliations:** Department of Biochemistry, The University of Mississippi Medical Center, Jackson, MS 39216-4505, USA

**Keywords:** Cajal body, Coilin, snRNA

## Abstract

Spliceosomal small nuclear ribonucleoproteins (snRNPs) are enriched in the Cajal body (CB). Guide RNAs, known as small Cajal body-specific RNAs (scaRNAs), direct modification of the small nuclear RNA (snRNA) component of the snRNP. The protein WRAP53 binds a sequence motif (the CAB box) found in many scaRNAs and the RNA component of telomerase (hTR) and targets these RNAs to the CB. We have previously reported that coilin, the CB marker protein, associates with certain non-coding RNAs. For a more comprehensive examination of the RNAs associated with coilin, we have sequenced the RNA isolated from coilin immunocomplexes. A striking preferential association of coilin with the box C/D scaRNAs 2 and 9, which lack a CAB box, was observed. This association varied by treatment condition and WRAP53 knockdown. In contrast, reduction of WRAP53 did not alter the level of coilin association with hTR. Additional studies showed that coilin degrades/processes scaRNA 2 and 9, associates with active telomerase and can influence telomerase activity. These findings suggest that coilin plays a novel role in the biogenesis of box C/D scaRNPs and telomerase.

## INTRODUCTION

Cajal bodies (CBs) were first reported by Ramon y Cajal in neuronal cells as “nucleolar accessory bodies” because both the nucleolus and CBs were visible by the same staining technique (Ramón y Cajal, 1903). CBs are enriched or associated with factors required for small nuclear ribonucleoprotein (snRNP) biogenesis (Machyna et al., 2013), telomerase biogenesis (Zhu et al., 2003), ribosomal RNA (rRNA) biogenesis (Isaac et al., 1998; Raška et al., 1990), and RNA transcription and processing (Cioce and Lamond, 2005; Gall, 2000). Coilin is the protein marker for CBs and required for CB integrity (Hebert, 2010). Reduction of coilin by knockdown or knockout causes the disassembly of CBs and decreased cell proliferation and organismal viability in mouse and zebrafish but not in *Drosophila* (Lemm et al., 2006; Liu et al., 2009; Strzelecka et al., 2010; Tucker et al., 2001; Walker et al., 2009). Other proteins enriched in the CB are the survival of motor neuron (SMN) protein, which is mutated in most cases of spinal muscular atrophy (Coady and Lorson, 2011), and WRAP53 (also known as TCAB1 or WDR79), which plays a pivotal role in RNP biogenesis (Tycowski et al., 2009; Venteicher et al., 2009; Mahmoudi et al., 2010; Stern et al., 2012).

Certain RNAs are enriched in CBs, including U snRNAs (Carmo-Fonseca et al., 1993; Carmo-Fonseca et al., 1992; Carmo-Fonseca et al., 1991b; Carmo-Fonseca et al., 1991a; Matera and Ward, 1993), small Cajal body-associated RNAs (scaRNAs) (Richard et al., 2003), and the telomerase RNA component (hTERC/hTR) (Zhu et al., 2003), likely reflecting the role CBs play in both snRNP and telomerase biogenesis. In addition to mature snRNAs, pre-processed snRNAs are also found in the CB (Smith and Lawrence, 2000), along with protein components necessary for the co-transcriptional processing of these snRNAs (Takata et al., 2012). CBs associate with specific gene loci including those encoding certain histones and U snRNAs (Frey and Matera, 1995). It is believed that CBs associate with histone gene loci in order to provide factors, such as the U7 snRNP, that are necessary for histone 3′ end processing. Somewhat confusingly, the U7 snRNP and other proteins such as FLASH and NPAT are also found, in other organisms such as *Drosophila*, in another subnuclear domain known as the histone locus body (HLB) (Liu et al., 2006; Nizami et al., 2010a; Nizami et al., 2010b). HLBs are associated with the processing of histone pre-mRNA transcribed during S phase. In human cancer cells, however, the U7 snRNP accumulates in CBs and not HLBs, but CBs and HLBs share other common components, such as coilin (Frey and Matera, 1995; Rajendra et al., 2010). Coilin forms a complex with U2 snRNA, ribosomal RNA (rRNA) and hTERC in cells, and chromatin immunoprecipitation (ChIP) experiments show that coilin associates with specific coding regions of the U1 and U2 snRNA gene loci (Broome and Hebert, 2013). In addition, knockdown and overexpression studies have shown that significant changes in the steady state levels of certain U snRNAs, rRNA and hTERC occur as a result of altered coilin levels (Broome et al., 2013; Broome and Hebert, 2013).

We have previously shown that coilin has RNase activity *in vitro* and can cleave the precursor transcripts of both U2 snRNA and hTERC (Broome et al., 2013; Broome and Hebert, 2012; Broome and Hebert, 2013), which supports the idea of coilin involvement in the processing of these RNAs. Other studies have found that coilin can form a complex with WRAP53 (Mahmoudi et al., 2010), but it is not known if this interaction is direct. WRAP53 interacts with a conserved sequence motif (the CAB box) present in many scaRNAs (Richard et al., 2003) and hTERC/hTR (Jády et al., 2004) and targets these RNAs to the CB (Tycowski et al., 2009; Venteicher et al., 2009; Mahmoudi et al., 2010; Stern et al., 2012). At the CB the scaRNA binds proteins forming a scaRNP, which then directs the direct modification of the snRNA component of the snRNP by 2′-*O*-methylation and pseudouridylation. There are three distinct classes of scaRNAs containing i) box C/D motifs that guide 2′-*O*-methylation, ii) box H/ACA sequences that direct pseudouridylation, or iii) mixed domain scaRNAs that guide both types of modifications. In human, there are at least 24 different scaRNAs (Lestrade and Weber, 2006). Four of these are strictly box C/D, four are mixed domain and the rest are box H/ACA. Interestingly, human box C/D scaRNAs lack a consensus CAB motif. A CAB-like motif, however, is present in *D. melanogaster* box C/D scaRNAs and the fly homologue of WRAP53 can be crosslinked to this sequence (Tycowski et al., 2009). In contrast, human WRAP53 fails to crosslink with *Drosophila* C/D CAB-like box-containing stemloops, which suggests that, in human, WRAP53 binds another sequence within the C/D scaRNAs apart from the CAB box. In support of this idea, human C/D scaRNAs are recovered from human WRAP53 immunoprecipitation complexes (Tycowski et al., 2009). Alternatively, it is possible that C/D scaRNAs in human do not directly interact with WRAP53 but are found in the WRAP53 immunoprecipitation complex via interactions with another protein found in complex. One possible candidate for this C/D scaRNA-interacting protein is coilin, which is present in the WRAP53 immunoprecipitation complex (Mahmoudi et al., 2010) and associates with other non-coding RNAs such as hTERC/hTR (Broome et al., 2013; Broome and Hebert, 2013).

To more fully examine the repertoire of RNAs that associate with coilin, we have isolated and sequenced the RNA recovered from coilin immunocomplexes from HeLa cells after different treatment conditions. Several non-coding RNAs, including hTERC/hTR, were enriched in the complexes. Notably, the box C/D scaRNAs 2 and 9 were, by a substantial margin, the most abundant non-coding RNAs recovered in the coilin immunocomplexes. Additional experiments were conducted to determine if WRAP53 and coilin directly interact and ascertain if WRAP53 mediates the association of coilin with specific non-coding RNAs. *In vitro* studies using purified coilin demonstrate that coilin can specifically process scaRNA 9. Given that coilin associates with hTERC/hTR, and may possibly play a role in its processing, we also examined if coilin is associated with telomerase or can modulate telomerase activity. Taken together, the results presented here further implicate a role for the CB marker protein in telomerase biogenesis and strongly suggest a novel function for coilin in the formation of box C/D scaRNPs.

## RESULTS

### Association of coilin with scaRNAs

In human, only box H/ACA or mixed domain scaRNAs contain CAB boxes (Lestrade and Weber, 2006; Richard et al., 2003). In contrast, box C/D scaRNAs (2, 7, 9, 17) do not contain a CAB box in the appropriate context. It is therefore unclear how these RNAs are targeted to the CB and incorporated into scaRNPs. Although *Drosophila* C/D scaRNAs contain a CAB-like motif that interacts with the *Drosophila* homologue of WRAP53 (Tycowski et al., 2009), this relationship does not appear to be conserved in human. Human box C/D scaRNAs can be found in WRAP53 immunocomplexes, but only in significant amounts when cells are lysed in stringent conditions (Tycowski et al., 2009). It is interesting to note that coilin requires stringent conditions for its extraction (Velma et al., 2012), and these conditions were utilized in order to show an association of box C/D scaRNAs with WRAP53 (Tycowski et al., 2009). Based on the above findings, we hypothesized that WRAP53 indirectly interacts with box C/D scaRNAs and another *trans* factor besides WRAP53 is directly responsible for the localization of these scaRNAs to the CB. Given that coilin can form a complex with WRAP53 (Mahmoudi et al., 2010) and associates with another scaRNA, hTERC/hTR (Broome et al., 2013; Broome and Hebert, 2013), we further hypothesized that coilin is this other *trans* factor.

To test these hypotheses, we identified the RNAs found in human (HeLa) coilin immunocomplexes by RNA sequencing. In addition to untreated cells, lysate was generated from cells treated with etoposide, actinomycin D or nocodazole. These conditions impact coilin localization and phosphorylation (Carmo-Fonseca et al., 1993; Carmo-Fonseca et al., 1992; Velma et al., 2012). Enrichment of RNA in the coilin immunocomplexes was relative to that found in control IgG immunocomplexes for each condition. In the untreated condition, 6 of the top 9 most enriched RNAs present in the coilin complex are scaRNAs (in order: 2, 9, 13, hTERC/hTR, 17, and 10). The other three RNAs are small nucleolar RNAs. The complete set of RNAs enriched on coilin complexes from each of the different conditions is shown in supplementary material Tables S1–S4. The most abundant scaRNAs obtained from coilin complexes from untreated cells is shown in Table 1. Additionally, the fold enrichment of scaRNA 12 is also shown. Table 1 also contains the fold enrichment of these same RNAs after etoposide (DNA damage), actinomycin D (transcription inhibitor) or nocodazole (arrests cells in mitosis) treatment. Strikingly, the box C/D scaRNAs 2 and 9 are enriched 81-fold and 77-fold, respectively, in coilin complexes from untreated cells compared to control IgG complexes. The next most abundant RNA is scaRNA 13 (box H/ACA), with a 23-fold increase, followed by hTERC/hTR. We have previously reported by quantitative reverse transcriptase real-time PCR a 17-fold enrichment of hTERC/hTR in coilin complexes (Broome and Hebert, 2012), so the RNA sequencing data presented here (showing a 21-fold increase) agree with those results. The final two most abundant scaRNAs (10 and 12) are mixed domain scaRNAs that contain both box C/D and box H/ACA domains.

**Table 1.**
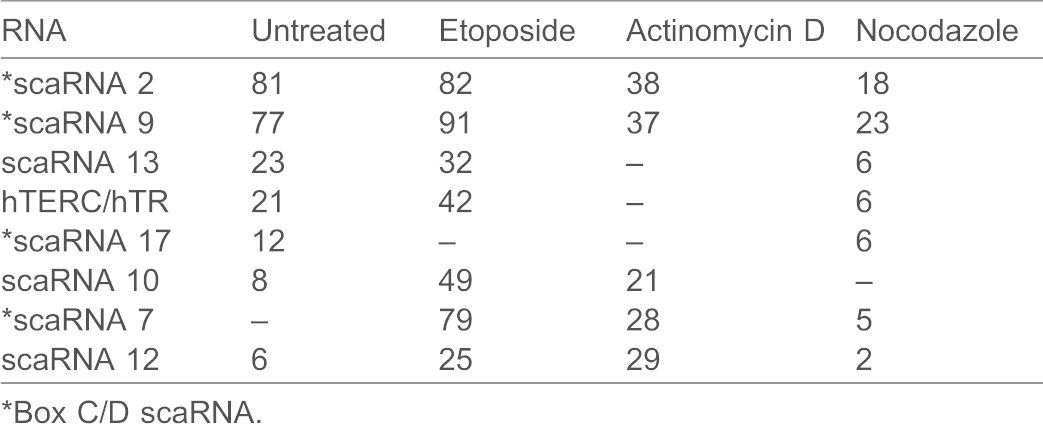
Fold enrichment from coilin IP relative to IgG IP for each condition

When comparing the abundance of RNAs found in the coilin complex across the different treatments tested, box C/D scaRNA 2 and 9 are consistently the most enriched (Table 1). The other two box C/D scaRNAs, 17 and 7, are more varied in their enrichment in the coilin complex. For example, scaRNA 17 is enriched 12-fold in untreated cells, 6-fold after nocodazole treatment but was not detected in coilin immunocomplexes following etoposide or actinomycin D exposure (Table 1). In contrast, scaRNAs 7 is not detected in untreated cells and only 5-fold enriched in after nocodazole treatment, but is highly enriched after etoposide (79-fold) and actinomycin D (28-fold) treatments. Although scaRNA 2 and 9 are still the most abundant RNAs enriched in the coilin complexes after actinomycin D and nocodazole treatment, this enrichment was attenuated. Furthermore, nocodazole treatment, which causes cell cycle arrest in mitosis and hyperphosphorylated coilin, results in the lowest amount of scaRNA 2 and 9 recovered, as well as a decreased amount of the other scaRNAs.

### The RNA binding domain of coilin directly interacts with WRAP53

**Fig. 1.**
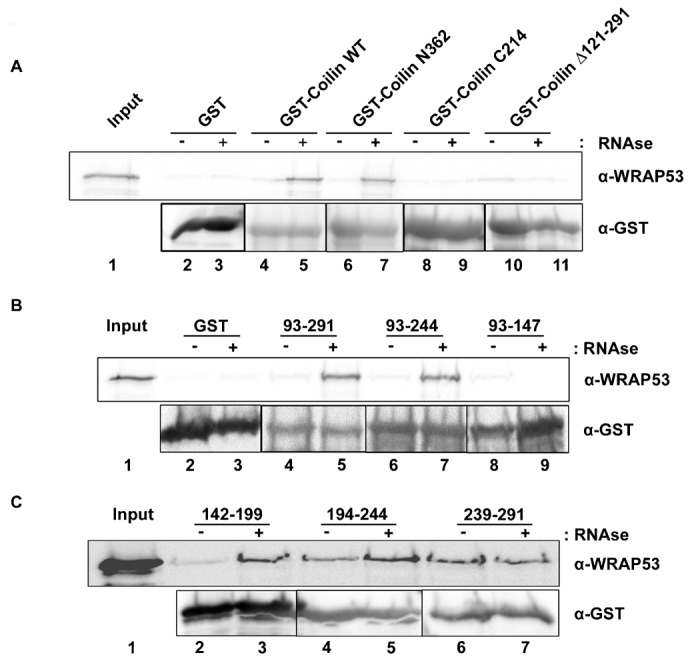
Bacterial RNA inhibits direct coilin interaction with WRAP53. (A–C) GST-pulldown experiments were conducted using GST, GST-coilin (or fragments thereof) and His-T7-tagged WRAP53 partially purified from bacteria. Pulldown reactions were subjected to SDS-PAGE, Western blotting, and probing with the indicated antibodies. RNase A/T1 treated GST proteins were also used (+). The input lane represents 10% of the His-T7-WRAP53 used in each reaction.

Human coilin consists of 576 amino acids (aa). We have previously found that coilin residues 121–291, which lie within a region of predicted disorder (Makarov et al., 2013), contribute greatly to coilin nucleic acid binding and RNA degradation activities (Broome and Hebert, 2013). A previous report has shown that coilin and WRAP53 can form a complex (Mahmoudi et al., 2010), but it is not known if this association is direct. To test for direct interaction, bacterially purified coilin (or fragments thereof) and WRAP53 were used in GST-pulldown assays (Fig. 1). A potential complication for these experiments is the fact that bacterially isolated coilin co-purifies with a large amount of nucleic acid, most of which is RNA (Broome and Hebert, 2012). Hence the binding of bacterial RNA to coilin may interfere with the ability of WRAP53 to interact with coilin. To overcome this obstacle, reactions were conducted using GST protein untreated or pre-treated with RNase A/T1 to remove co-purifying bacterial RNA. As shown in Fig. 1A, the amount of WRAP53 recovered by untreated full-length coilin (WT), N-terminal 1–362 aa (N362), C-terminal 362–576 (C214) or the 121–291 deletion is not significantly greater than that recovered by GST alone (compare lane 2 with lane 4, 6, 8 and 10). However, RNase A/T1 pre-treatment of the GST proteins resulted in a clear recovery of WRAP53 over WT and N362 coilin beads (lanes 5 and 7). Even with RNase A/T1 treatment, the C214 fragment failed to bind WRAP53 (lane 9). Likewise, the 121–291 deletion did not recover significant amounts of WRAP53, regardless of RNase A/T1 treatment (lanes 10 and 11). These findings demonstrate that WRAP53 directly interacts with coilin in the general region of coilin that binds nucleic acid (121–291), and RNA inhibits the binding of WRAP53 to coilin. To further delimit the binding site on coilin for WRAP53, six additional coilin fragments, spanning aa 93–291, were tested. As before, these GST-coilin fragments were tested with or without RNase A/T1 pre-treatment (Fig. 1B,C). No interaction of WRAP53 was observed for coilin fragment 93–147, regardless of RNase A/T1 treatment (Fig. 1B, lanes 8 and 9). In contrast, coilin fragment 239–291 recovered WRAP53, and RNase A/T1 treatment did not have any impact on this recovery (Fig. 1C, lanes 6 and 7). The other four fragments tested (93–291, 93–244, 142–199 and 194–244) all showed increased association with WRAP53 after pre-treatment with RNase A/T1 (Fig. 1B,C). In conclusion, of the six coilin fragments tested, only 93–147 fails to recover WRAP53. Hence WRAP53 directly interacts with coilin via residues 148–291, which includes the coilin RNA binding domain.

### Coilin and WRAP53 associations with hTERC/hTR are not interdependent

**Fig. 2.**
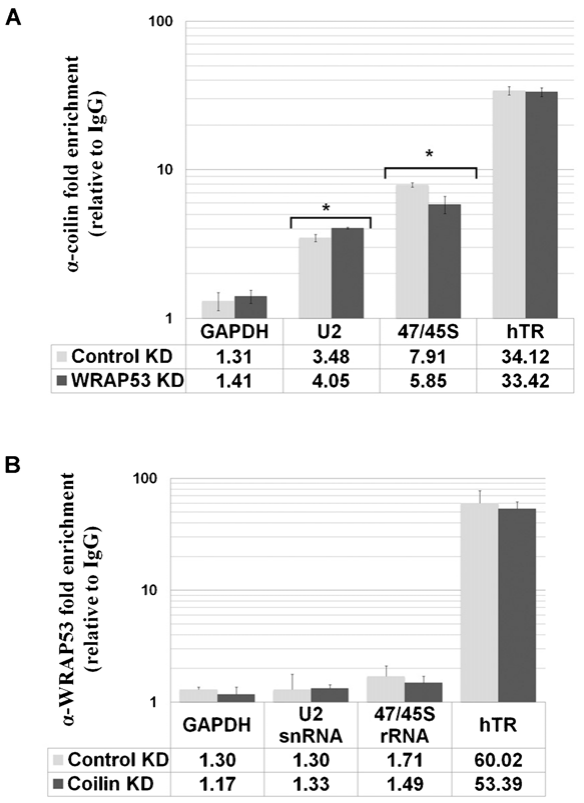
Coilin and WRAP53 associations with hTERC/hTR are not interdependent. (A) HeLa cells were transfected with control or WRAP53 siRNA. 48 h post transfection, RNA IPs were performed using IgG or α-coilin antibodies, the RNA isolated and analyzed by qRT-PCR. RNA enrichment from the coilin IP was normalized to IgG IP and presented in the histogram. (B) HeLa cells were transfected with control or coilin siRNA. 48 h post transfection, RNA IPs were performed using IgG or α-WRAP53 antibodies, the RNA isolated and analyzed by qRT-PCR. The RNA enrichment from WRAP53 IP was normalized to IgG IP and presented in the histogram. Error bars represent 1 s.d. from 2 experimental repeats and 3 technical repeats; *p<0.005 relative to control KD IP for each RNA.

We have shown that coilin specifically interacts with a number of non-coding RNAs, such as hTERC/hTR, as determined by quantitative reverse transcriptase real time PCR using RNA isolated from coilin immunocomplexes (Broome et al., 2013; Broome and Hebert, 2013). These findings were corroborated by the RNA sequencing results presented above (Table 1). Since WRAP53 is known to bind the CAB motif present in hTERC/hTR (Venteicher et al., 2009; Tycowski et al., 2009), and associate with coilin (Mahmoudi et al., 2010; this study), it is possible that hTERC/hTR in the coilin complexes is indirect and mediated by WRAP53. Alternatively, it is also possible that coilin, or another protein in the coilin complex, directly interacts with hTERC/hTR and WRAP53 does not impact this association. To test these two possibilities, RNA immunoprecipitation (IP) was performed using HeLa lysate with control (IgG), α-coilin or α-WRAP53 antibodies following knockdown of either coilin or WRAP53 (Fig. 2). WRAP53 and coilin knockdown was verified by Western blotting (supplementary material Fig. S1A,B). The RNA isolated from the IP beads was subjected to reverse transcriptase real-time PCR using primers for hTERC/hTR, as well as 47/45S rRNA and U2 snRNA for comparison, since we have previously published an association between coilin and these RNA transcripts (Broome and Hebert, 2013). GAPDH message was used as a non-CB related control. As expected, coilin associates with a significant amount of hTERC/hTR in the control knockdown treatment, and also enriches for U2 snRNA and 47/45S pre-rRNA (Fig. 2A). Reduction of WRAP53 did not impact hTR association with coilin, suggesting that WRAP53 is not bridging the coilin-hTR interaction (Fig. 2A). However, U2 and 47/45S RNA association with coilin is slightly altered upon WRAP53 knockdown. The reciprocal experiment, in which coilin was reduced and the amount of co-immunoprecipitated hTERC/hTR from WRAP53 IP beads was determined, reveals that the level of hTR in the WRAP53 complex does not change upon coilin knockdown (Fig. 2B). These results demonstrate that hTERC/hTR association with WRAP53 or coilin is not interdependent.

### Box C/D scaRNA 2 and 9 abundance in coilin and WRAP53 immunocomplexes

**Fig. 3.**
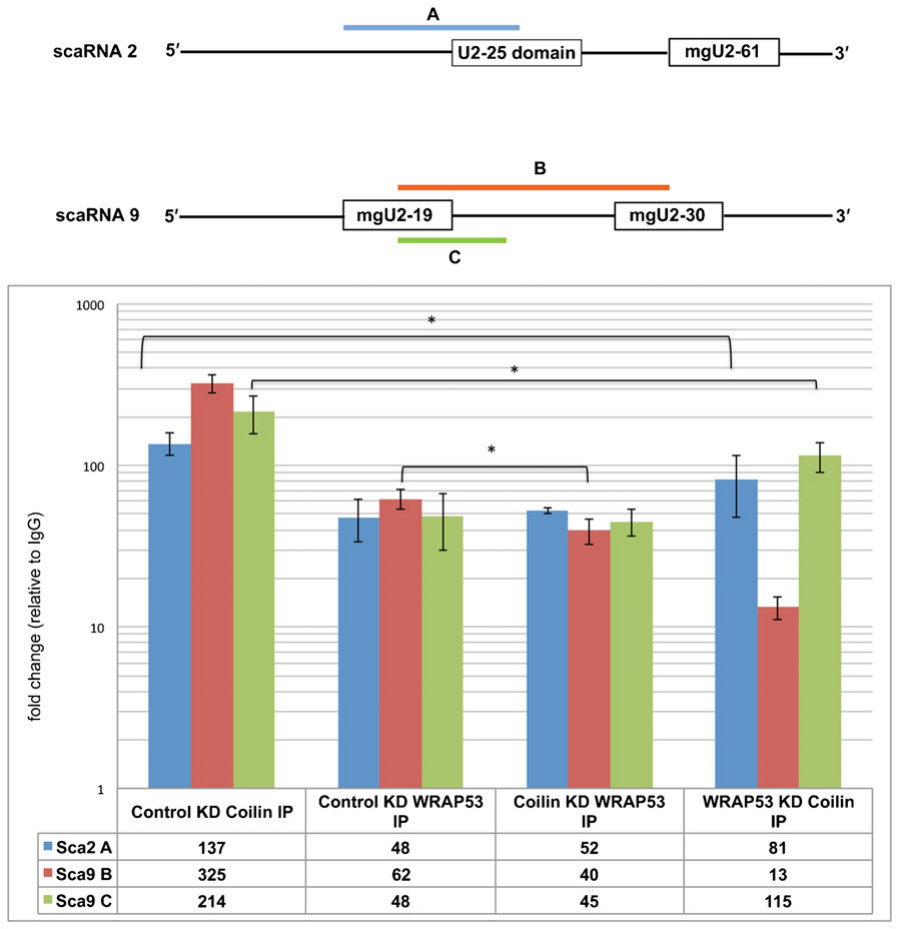
WRAP53 reduction decreases the enrichment of scaRNA 2 and 9 in the coilin complex. Schematic of scaRNA 2 and scaRNA 9 showing regions to be amplified by qPCR. HeLa cells were transfected with control, coilin or WRAP53 siRNA. 48 h post transfection, RNA IPs were performed using IgG, α-WRAP53 or α-coilin antibodies, the isolated RNA was converted to cDNA and analyzed by qPCR. The amplicon amounts from WRAP53 IP and coilin IP reactions were normalized (fold change) to that obtained for control IgG IP for each of the siRNA conditions (histogram). Error bars represent 1 s.d. from 3 experimental repeats; *p<0.005 relative to control knockdown (KD) IP for each RNA.

The RNA sequencing results described above (Table 1) show that coilin immunocomplexes are greatly enriched for the box C/D scaRNAs 2 and 9. To examine if WRAP53 may be mediating this enrichment, IPs were conducted using lysate generated from HeLa cells transfected with control, coilin or WRAP53 siRNA, followed by RNA isolation, cDNA generation and qPCR using primers specific to scaRNA 2 and 9 (Fig. 3). Both scaRNA 2 and 9 are processed to yield smaller fragments that guide modifications on U2 snRNA (Tycowski et al., 2004). For scaRNA 2, mgU2-61 is generated while two guide RNAs are processed from scaRNA 9: mgU2-19 and mgU2-30. Primer sets for scaRNA 9 were used to examine if coilin complexes are associated with unprocessed scaRNA 9 (amplicons B and C). As observed for the RNA sequencing results, coilin complexes are highly enriched for both scaRNA 2 (amplicon A) and scaRNA 9 (amplicons B and C) in control siRNA treated cells. We have found that WRAP53 complexes are also enriched for these two RNAs, and the reduction of coilin did not change the amount of amplicon A or C. However, a reduction was observed for scaRNA 9 (amplicon B) in WRAP53 complexes upon coilin knockdown. Upon knockdown of WRAP53 and IP with coilin, the amount of scaRNA 2 and 9 amplicons was reduced compared to control siRNA treated cells but still highly enriched. Strikingly, the amount of amplicon B, which spans the two scaRNA 9 processed regions (mgU2-19 and mgU2-30) was greatly decreased in the coilin complex after WRAP53 knockdown (from 325-fold to 13-fold). Considering that coilin has RNase activity and can specifically degrade U2 snRNA and hTERC/hTR *in vitro*, it is possible that the reduced enrichment of scaRNA 9 amplicon B in coilin complexes after WRAP53 knockdown is indicative of a regulatory role for WRAP53 in controlling coilin-mediated processing of scaRNA 9.

### *In vitro* degradation/processing of scaRNA 2 and 9 by coilin

**Fig. 4.**
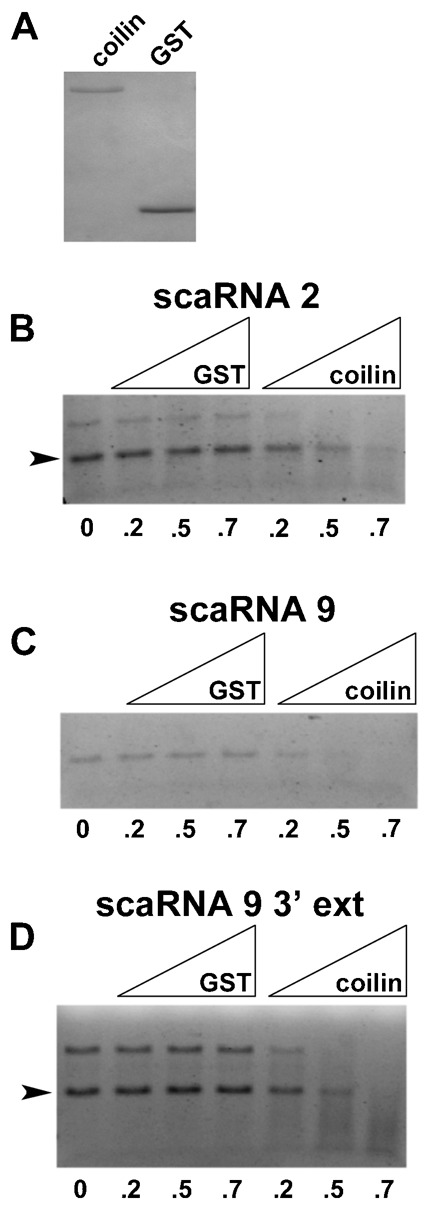
Purified coilin degrades scaRNA 2 and 9 *in vitro*. (A) Coilin and GST, purified to homogeneity, were subjected to SDS-PAGE and the gel was Coomassie stained. (B–D) RNA degradation assays using purified coilin or GST, shown in panel A, with 100 nM *in vitro* transcribed scaRNA 2 (B), scaRNA 9 (C) or scaRNA 9 containing an extended 3′-end (D). RNAs were incubated without protein (0) or with increasing amounts (approximately 0.2 µg, 0.5 µg and 0.7 µg) of GST or coilin. Reactions were then subjected to agarose gel electrophoresis and ethidium bromide staining. The arrowheads in panels B and D demarcate the full-length scaRNA based on expected size.

To examine if scaRNA 2 and 9 are substrates for the RNase activity of coilin, *in vitro* transcribed scaRNA 2, scaRNA 9 and scaRNA 9 with a 3′ extension were generated. These RNAs were incubated with purified coilin or GST (Fig. 4A) and degradation/processing of the RNA was monitored by agarose gel electrophoresis. Compared to the reactions lacking protein (0), little degradation of the *in vitro* transcribed scaRNAs is observed in the presence of GST (Fig. 4B–D). However, the amount of full-length scaRNAs is reduced upon the addition of coilin. Thus scaRNA 2 and 9 are substrates for the RNase activity of coilin.

**Fig. 5.**
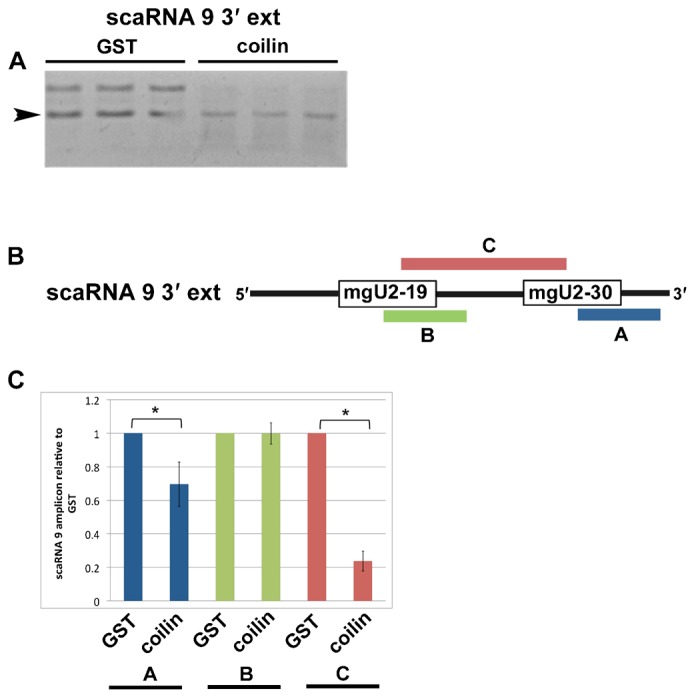
Coilin specifically processes scaRNA 9. (A) *In vitro* transcribed scaRNA 9 with a 3′ extension (100 nM) was incubated with 300 ng of purified GST or coilin (shown in Fig. 4A). Triplicate reactions were conducted. The arrowhead marks the position of the full-length scaRNA 9 with 3′ extension based on expected size. (B) Schematic of scaRNA 9 3′ extension showing regions to be amplified by qRT-PCR. (C) RNA degradation reactions shown in panel A were analyzed by qRT-PCR. The products from the coilin reactions were normalized to that obtained from the GST reactions and presented in the histogram. Error bars represent 1 s.d. from 3 experimental repeats; *p<0.005 relative to product amount in the GST reactions for indicated amplicon.

It is known that scaRNA 2, 9 and 17 are processed to generate smaller fragments (Tycowski et al., 2004), but how this is accomplished is unclear. Since we have found that coilin associates with and can degrade scaRNA 9, we next examined if coilin shows specificity in its RNase activity towards this RNA. For these studies, 300 ng of purified GST or coilin was incubated with 100 nM scaRNA 9 containing a 3′ extension for 30 min at 37°C, followed by DNase treatment. Reactions were conducted in triplicate and subjected to agarose gel electrophoresis and quantitative reverse transcriptase real-time PCR (Fig. 5). As observed previously (Fig. 4D), coilin, but not GST, addition results in a decrease in the amount of scaRNA 9 3′ extension (Fig. 5A). To monitor if the RNA degradation activity of coilin is specific to certain regions of the scaRNA 9 substrate, the same reactions shown in Fig. 5A were used for quantitative reverse transcriptase real-time PCR with primers shown in Fig. 5B. These primer pairs were designed to examine scaRNA 9 3′-end processing (amplicon A), mgU2-19 generation (amplicon B) and cleavage of the region between mgU2-19 and mgU2-30 (amplicon C). This analysis is shown in Fig. 5C and the data for the coilin reactions are normalized to GST for each primer pair. In the presence of coilin, the amount of scaRNA 9 3′-end (amplicon A) is significantly reduced, yet the amount of mgU2-19 is not changed (amplicon B). Most dramatically, the amount of amplicon C is reduced nearly 80% in reactions containing coilin versus GST, indicating that coilin has specificity in degrading the region between mgU2-19 and mgU2-30.

### Coilin expression in a coilin knockout background increases telomerase activity

**Fig. 6.**
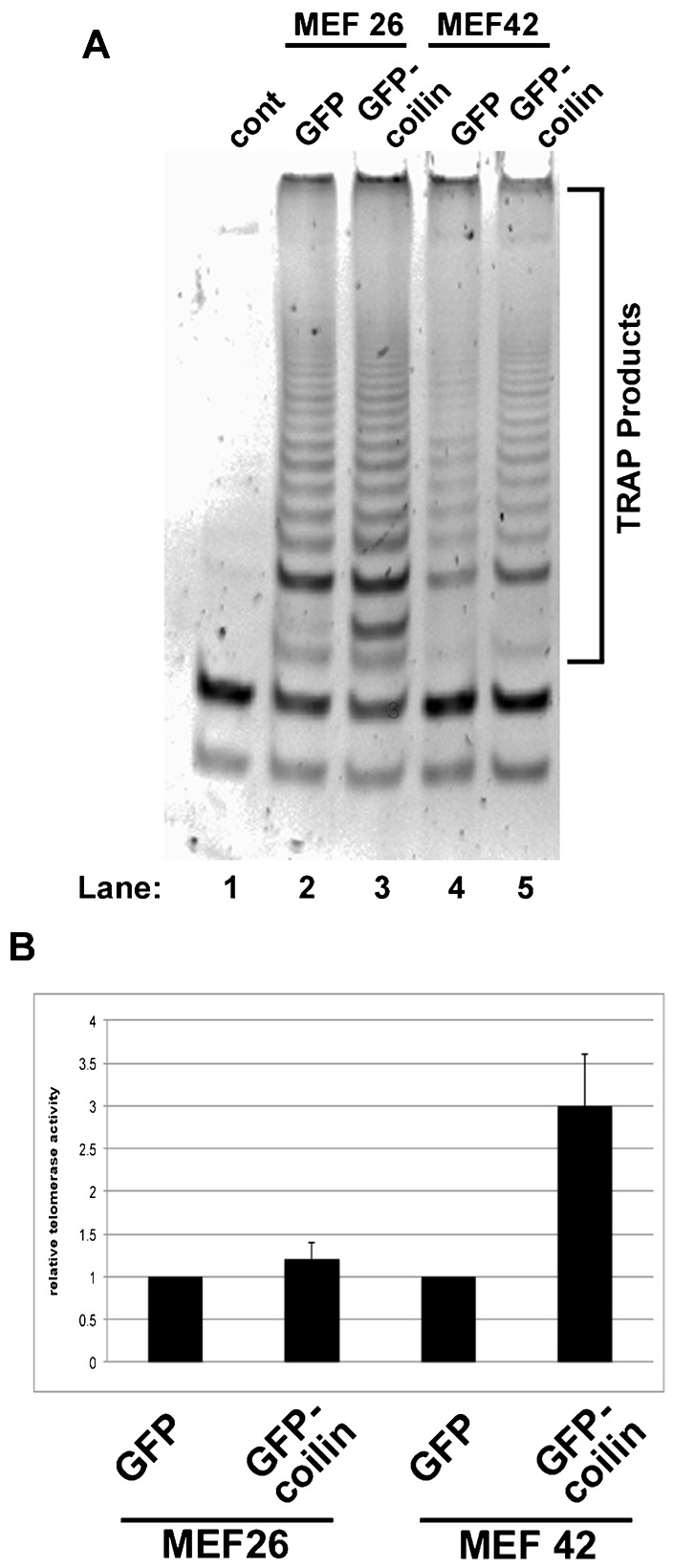
Coilin increases telomerase activity in a coilin knockout background. (A) MEF26 (WT) and MEF42 (coilin knockout) cells were transfected with GFP only or GFP-mouse coilin. Cell lysates were used in TRAP assays, and the products separated by PAGE and visualized by ethidium bromide staining. (B) The TRAP products were quantified by densitometry and normalized to the GFP values for each cell line. Error bars represent 1 s.d. from three separate experiments. In MEF42 cells, the TRAP products are significantly lower in GFP expressing cells compared to GFP-mouse coilin expressing cells (p<0.05).

The RNA sequencing results (Table 1) and our previous studies (Broome et al., 2013; Broome and Hebert, 2013) show that coilin associates with hTERC/hTR. Moreover, we have shown that coilin RNA degradation activity has specificity towards the 3′-end of pre-hTERC/hTR (Broome et al., 2013; Broome and Hebert, 2013). These findings suggest that coilin participates in the biogenesis of telomerase. To test this hypothesis, we examined the activity of telomerase in lysates from mouse cell lines with (MEF26) or without (MEF42) coilin expression. It should be noted that the coilin knockout cell line (MEF42) is not a complete knockout, but has the potential to encode a N-terminal fragment of the protein, or about 15% of the full-length protein (Tucker et al., 2001). Both wild-type (MEF26) and coilin knockout (MEF42) lines were transfected with GFP vector only or GFP-mouse coilin DNA for 24 hrs followed by lysate generation and measurement of telomerase by a PCR based telomeric repeat amplification protocol (TRAPeze assay). TRAP products were analyzed by polyacrylamide gel electrophoresis (PAGE) and visualized by ethidium bromide staining. A representative gel is shown in Fig. 6A, and a histogram of the quantification of the TRAP products for three experiments is shown in Fig. 6B. In the wild-type (MEF26) background, the expression of GFP-coilin does not change the activity of telomerase relative to that obtained from cells expressing GFP only. In contrast, GFP-coilin expression in the coilin knockout (MEF42) background significantly increases telomerase activity approximately 3-fold. These findings thus further support the hypothesis that coilin is involved in telomerase biogenesis.

### Coilin associates with active telomerase in HeLa cells

**Fig. 7.**
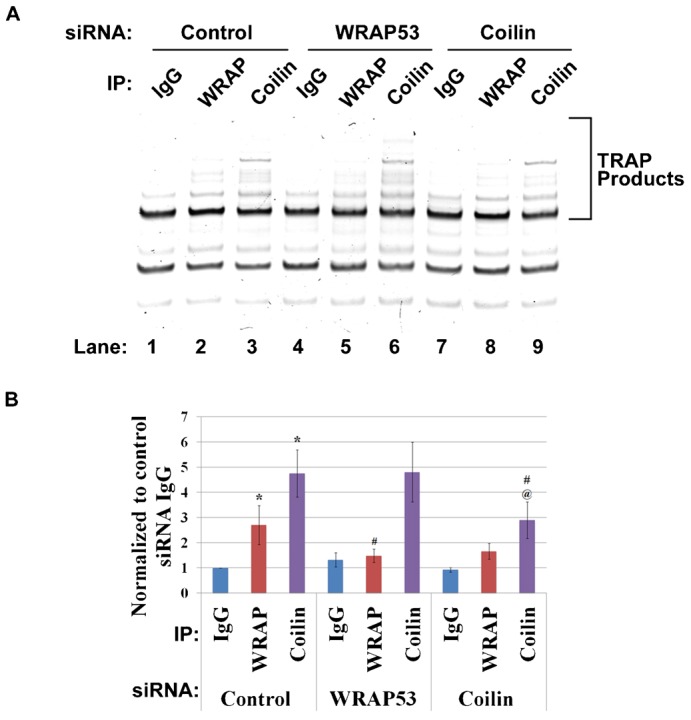
Coilin associates with active telomerase in HeLa cells. (A) HeLa cells were transfected with control, WRAP53 or coilin siRNA. 48 h post transfection, cell lysates were subjected to IP with IgG, α-WRAP53 or α-coilin antibodies. IP complexes were used in TRAP assays, and the products separated by PAGE and visualized by ethidium bromide staining. (B) The TRAP products were quantified by densitometry and normalized to control knockdown IgG IP. Error bars represent 1 s.d. from three independent experiments. *p<0.05 relative to that obtained for control siRNA treated lysate IPed with control IgG. ^#^p<0.05 relative to control siRNA of respective IP. ^@^p<0.05 relative to WRAP53 siRNA lysate subjected to coilin antibody IP.

In light of the evidence that both coilin and WRAP53 independently associate with hTERC (Fig. 2), we wanted to investigate the association between WRAP53 and active telomerase following coilin knockdown. In addition, due to the association between coilin and hTERC, we hypothesized that coilin would also associate with active telomerase. To examine the association between active telomerase and WRAP53 or coilin, we performed immunoprecipitation with lysates following control, WRAP53 or coilin siRNA transfection, and then measured the activity of associated telomerase (TRAPeze assay). Control (IgG), WRAP53 and coilin antibodies were used for the immunoprecipitations. TRAP products were analyzed by polyacrylamide gel electrophoresis (PAGE) and visualized by ethidium bromide staining (Fig. 7A, quantification in Fig. 7B). Negative control reactions using heat inactivation (85°C for 10 min) or RNase treatment significantly decreased the amount of TRAP products. Quantification of the TRAP products from three separate experiments is shown in Fig. 7B, normalized to that obtained from the IgG IP of control siRNA treated cells. Telomerase activity is most abundant in the coilin IP reactions, a somewhat unanticipated result. As expected, there is a significant decrease in coilin associated telomerase activity following coilin knockdown (Fig. 7A, compare the intensity of the TRAP product signals in lane 3 and 6 to that in lane 9, quantified in Fig. 7B). Regarding the WRAP53 IP samples, telomerase activity is greatest when using control siRNA treated lysate to IP (Fig. 7A, lane 2). There is a significant decrease in telomerase activity in WRAP53 IPs when using WRAP53 reduced lysate (Fig. 7A, lane 5). The amount of TRAP products in WRAP IP with coilin knockdown lysate (Fig. 7A, lane 8) relative to that obtained from WRAP IP with control knockdown lysate (Fig. 7A, lane 2) is reduced, but not statistically significant (p = 0.052). These results suggest that there is an association between coilin and active telomerase that is not dependent upon WRAP53. To confirm the specificity of the IPs and the efficacy of the knockdowns, the same beads used for the TRAP assay in Fig. 7 were subjected to SDS-PAGE and Western blotting (supplementary material Fig. S2A). Note that the amount of immunoprecipitated WRAP53 (lane 5) and coilin (lane 9) is reduced upon treatment with WRAP53 or coilin siRNA, respectively. Also note that coilin is co-immunoprecipitated with WRAP53 in control and coilin siRNA treated cells (faint coilin bands in lane 2 and 8), in agreement with a previous report (Mahmoudi et al., 2010).

## DISCUSSION

### Coilin: a scaRNA associated protein

Recent work indicates that coilin may have other roles apart from serving as a structural component of the CB. For example, centromeres damaged by infection with herpes simplex virus accumulate coilin (Sabra et al., 2013; Morency et al., 2007) and coilin associates with specific RNA and DNA sequences in the cell (Broome and Hebert, 2013). Although considered the Cajal body marker protein, coilin is predominantly found in the nucleoplasm (Lam et al., 2002). Additionally, coilin is expressed in cells that lack or have few CBs (Young et al., 2000), suggesting that the activity of this protein is needed whether or not CBs are present. As part of our effort to understand the function of coilin both in the CB and the nucleoplasm, we have examined the RNAs associated with coilin immunocomplexes from human (HeLa) cells. In a previous study (Broome and Hebert, 2013), we employed a candidate approach using qRT-PCR and determined that coilin binds to 47/45S rRNA, U2 snRNA and hTERC/hTR. In this present study, we have employed RNA sequencing to better characterize all of the RNAs found in the coilin complex (Table 1; supplementary material Tables S1–S4). The majority of RNAs highly enriched in the coilin complex are scaRNAs, including hTERC/hTR. Noteworthy, scaRNA 2 and 9, which are box C/D scaRNAs that lack an obvious CAB motif, are by far the most abundant RNAs associated with coilin. We have previously observed that hTERC/hTR abundance in coilin complexes is reduced upon nocodazole treatment (Broome et al., 2013), and the RNA sequencing results reported here verify this finding. It remains to be determined if the reduction in associated RNAs found in response to nocodazole treatment can be directly attributed to the increased phosphorylation of coilin that takes place during mitosis.

### Coilin and 2′-*O*-methylation of U1 and U2 snRNA?

Upon examination of the enrichment data shown in Table 1, it is most curious that scaRNA 2 and 9 are so highly abundant, regardless of treatment condition, compared to the other associated RNAs (ranging from around 2 to 4 times more abundant than the next most abundant RNA). The only exception is the box C/D scaRNA 7 upon etoposide treatment, which is enriched to almost the same extent (79-fold) as scaRNA 2 and 9. Of all the RNAs in the cell, why are scaRNA 2 and 9 so highly enriched in the coilin complex? One possibility is that these two scaRNAs are the most abundant in HeLa cells. In our own limited analysis we have found that relative U1 snRNA levels are 4900-fold greater than hTR levels, and U2 snRNA levels are 1700-fold greater than hTR. Additionally, we have found that scaRNA 2 is more abundant than hTR, and hTR is more abundant that scaRNA 9, but none of these messages are as abundant as GAPDH (which is not associated with coilin). Furthermore, we observe that WRAP53 does not associate with scaRNA 2/9 to the same level as coilin. Thus it appears that coilin is specifically interacting with scaRNA 2/9 in the context of more abundant RNAs. The common characteristics shared between scaRNA 2 and 9 is that they are box C/D type scaRNAs that are subjected to processing (Tycowski et al., 2004). Interestingly, scaRNA 2 processing appears to be directed by the encoding region (Gérard et al., 2010), so it is possible that scaRNA 9 processing is likewise mediated by internal sequences. Our *in vitro* studies show that coilin can specifically degrade scaRNA 9 in the intervening region between mgU2-19 and mgU2-30 (Fig. 5), suggesting that coilin association with scaRNA 2 and 9 may contribute to their processing. Perhaps most importantly, scaRNA 2 and 9 are guide RNAs for the 2′-*O*-methylation of U2 snRNA. We have found previously that U2 snRNA is also enriched in the coilin complex and coilin associates with specific regions of the U2 snRNA gene (Broome and Hebert, 2013). We propose that coilin highly associates with scaRNA 2 and 9 in order to efficiently form their respective scaRNP particles. Since coilin appears to be tightly coupled to scaRNP 2, scaRNP 9 and U2 snRNP biogenesis, such an arrangement would be expected to greatly increase the rate of U2 snRNA 2′-*O*-methylation. Other factors, such as the poly(A) specific ribonuclease (PARN) that is found in nucleoli and CBs, likely also play a role (Berndt et al., 2012). A final point to consider in regards to the enrichment of scaRNAs in the coilin complex centers upon the dramatic increase in the amount of box C/D scaRNA 7 recovered in response to etoposide treatment (from not detected in untreated cells to 79-fold enriched in etoposide treated, Table 1). We have previously found that U1 snRNA is not detected in coilin complexes in untreated cells but is enriched (2.4-fold) after etoposide treatment, and coilin is associated with the U1 snRNA gene (Broome and Hebert, 2013). Since scaRNA 7 guides 2′-*O*-methylation of U1 snRNA, it is possible that the enrichment of both scaRNA 7 and U1 snRNA in coilin complexes in response to etoposide treatment serves to increase the efficiency of U1 snRNP biogenesis as a result of DNA damage.

### Differential impact of WRAP53 on coilin associated RNAs

Since WRAP53 has been shown to form a complex that can contain coilin (Mahmoudi et al., 2010), it is possible that WRAP53 mediates the interaction between coilin and the various RNAs identified in the coilin complex (Table 1; supplementary material Tables S1–S4). To explore this hypothesis, we first had to ascertain if WRAP53 and coilin directly interact, which has not been examined in previous studies. We show that WRAP53 and coilin do directly interact, and this interaction is mediated by coilin residues 148–291 (Fig. 1). Importantly, this region of coilin contains the RNA binding domain and RNase activity (Broome and Hebert, 2013). Bacterial RNA co-purified with GST-coilin (or fragments thereof) can inhibit the interaction of WRAP53 with coilin (Fig. 1). Collectively, these findings indicate that WRAP53 may regulate coilin interaction with, and degradation of, RNA.

Our work also shows that coilin association with hTERC/hTR is not mediated by interactions with WRAP53 (Fig. 2), and coilin is associated with active telomerase (Figs 6 and 7). These findings strongly argue for a role of coilin in telomerase biogenesis, possibly in the processing of the hTERC/hTR extended 3′ end primary transcript, and we have published data that support this idea (Broome and Hebert, 2013). A recent study has found that coilin is required for the recruitment of endogenous telomerase to telomeres (Stern et al., 2012). Although telomerase trafficking appears to differ in mice compared to human (Tomlinson et al., 2010), we propose that this requirement for coilin in human involves not only telomerase delivery, but also a direct involvement of coilin in the formation of this RNP.

Studies conducted to determine the relationship between WRAP53, coilin and scaRNA 2 and 9 reveal that these RNAs are more abundant in the coilin compared to the WRAP53 complex (Fig. 3). Furthermore, coilin reduction only modestly decreases the amount of one scaRNA 9 amplicon (amplicon B) in the WRAP53 complex. In contrast, WRAP53 reduction decreases the amount of scaRNA 2 and 9 associated with coilin immunocomplexes, although these RNAs are still highly enriched. Remarkably, the level of scaRNA 9 on coilin IP complexes detected by amplicon B, which spans the intervening region between mgU2-19 and mgU2-30, is reduced 25-fold (from 325 to 13) upon WRAP53 knockdown. Since the amount of scaRNA 9 amplicon C was decreased only 2-fold in coilin complexes isolated from WRAP53 siRNA cells as compared to control siRNA treated cells, we suspect that WRAP53 reduction results in an increase of coilin RNase activity targeted to the internal region of scaRNA 9. The exact nature of the relationship between coilin, WRAP53 and scaRNAs, especially as it relates to the possible regulation of coilin activity by WRAP53, awaits further study.

In summary, the results presented here further demonstrate a role for coilin in telomerase biogenesis. Our studies also uncover new functions for coilin centering upon box C/D scaRNP biogenesis, with WRAP53 as a possible regulator of these activities. It is unclear as to if coilin association with scaRNAs impacts its activity in HLBs, where this protein also accumulates. We speculate that there are three dynamic populations of coilin in the cell (nucleoplasmic, CB and HLB), and each of these groups is characterized by both protein and RNA interactions. Hence coilin in the CB would be expected to be more associated with scaRNAs compared to coilin in the HLB. Finally, the work presented here underscores why cancer cells invariably contain CBs: this subnuclear domain not only increases the efficiency of snRNP biogenesis but also may enhance scaRNP formation via coilin interactions with both proteins and scaRNAs. Both SMN and WRAP53 are induced in the transformation process, presumably to accommodate the increased demand for RNPs in cancerous cells (Mahmoudi et al., 2011; Sleeman et al., 2001). Like SMN and WRAP53, the expression of coilin is also increased in transformed cells compared to primary cells (supplementary material Fig. S2B), further indicating that coilin is part of the upregulated RNP biogenesis machinery present in cancer cells.

## MATERIALS AND METHODS

### Cell lines, cell culture, plasmids and transfections

HeLa and WI-38 lines were from the American Type Culture Collection (Manassas, VA, USA). MEF26 and MEF42 lines (Tucker et al., 2001) were from Greg Matera (University of North Carolina, Chapel Hill, NC, USA). Lines were cultured as described previously (Sun et al., 2005). GST-coilin WT, GST-coilin N362, GST-coilin C214 and GST-coilin Δ121–291 constructs were previously described (Broome and Hebert, 2013; Velma et al., 2010; Toyota et al., 2010; Hearst et al., 2009). His-tagged WRAP53, GST-coilin 93–291, GST-coilin 93–244, GST-coilin 93–147, GST-coilin 142–199, GST-coilin 194–244 and GST-coilin 239–291 constructs were made by PCR amplification using standard molecular biological techniques. GFP vector only and mouse GFP-coilin constructs were previously described (Shpargel et al., 2003). MEF26 and MEF42 cells were transfected with plasmid DNA using Lipofectamine 2000 transfection reagent (Invitrogen, Carlsbad, CA, USA) following the manufacturer's protocol. The non-targeting, control siRNA (Carrero et al., 2011) was obtained from Thermo Scientific (LaFayette, CO, USA). Coilin siRNA (N004645.12.4) (Toyota et al., 2010) and WRAP53 siRNA (N001143990.12.2) were obtained from Integrated DNA Technology (Coralville, IA, USA). SiRNAs were transfected into HeLa cells using Lipofectamine 2000 (Invitrogen, Carlsbad, CA, USA) per the manufacturer's suggested protocol.

### RNA sequencing

HeLa cells were untreated or treated with etoposide (20 µM) for 16 hrs, actinomycin D (2.5 µg/ml) for 2 hrs or nocodazole (0.4 µg/ml) for 16 hrs. RNA IPs were set up as described previously (Broome and Hebert, 2013) using control IgG (5 µg) or α-coilin (5 µg) antibodies. RNA was sequenced using Illumina TruSeq Stranded RNA LT kit (San Diego, CA, USA). The low sample protocol was used as per the manufacturer's instructions and the work was conducted by the UMMC Molecular and Genomics Facility. For the untreated samples, 4 independent IgG and coilin IP reactions were utilized. For the treated samples, 2 control IP and 2 coilin IP reactions were set up for each treatment condition. Data were analyzed using Gene Sifter, Analysis Edition, software (Perkin Elmer, Waltham, MA, USA).

### Cloning of scaRNA 2 and 9

ScaRNA 2, scaRNA 9 and scaRNA 9 with a 3′ extension were PCR amplified from HeLa genomic DNA. The following primers, some containing an *Eco*RI site (underlined), were used to PCR amplify scaRNA 2, scaRNA 9 and scaRNA9 3′ extension: (scaRNA 2) 5′-GCCGGATTCGTTTTAGGGAGGGAGAGCGGCCTG-3′ (forward), 5′-GGCGAATTCCCAGATCAGAATCGCCTCGATAAT-3′ (reverse); (scaRNA 9) 5′-GCCGGATTCCTTTCTGAGATCTGCTTTTAGTGA-3′ (forward), 5′-GGCGAATTCTGAGCTCAGGTCAAGTGTAGAAACC-3′ (reverse); and (scaRNA 9 3′ extension) 5′-GGCGAATTCAACAGTTGCTGAAGATAATGG-3′ (reverse). Products were cloned into the pCR4 TOPO TA cloning vector (Invitrogen, Carlsbad, CA, USA). The cloned product was digested with *Eco*RI, gel purified and cloned into pBluescript KS vector generating scaRNA 2, 9 and 9 with a 3′-end extension. All DNA constructs were sequence verified.

### *In vitro* transcription, RNA degradation/processing assays

ScaRNA 2 and scaRNA 9 clones in pBluescript KS were cut with *Bam*H1 and the scaRNA 9 3′ extension plasmid was cut with *Hind*III. The linearized vector was gel purified and the DNA template was used to transcribe *in vitro* scaRNA 2 and scaRNA 9 transcripts using T3 RNA polymerase and the Ambion MAXIscript *In vitro* Transcription Kit (Ambion Life Technologies, Carlsbad, CA, USA) as per the manufacturer's instructions. The same kit was used to produce the scaRNA 9 3′ extension transcript using T7 RNA polymerase. Pure, full-length nucleic acid free coilin was purified as previously described (Broome and Hebert, 2013), except that after electroelution the protein was put through an SDS removal column (Thermo Scientific, Rockford, IL, USA) previously washed with high salt (250 mM NaCl) PBS. GST was purified in a similar manner. RNA degradation assays were performed using purified GST and coilin. scaRNA 2, 9 and 9 3′ ext transcripts (100 nM) were incubated with no protein or increasing amounts (240 ng, 480 ng, 720 ng) of pure GST or coilin at 37°C for 30 min, followed by the addition of Turbo DNase (Ambion Life Technologies, Carlsbad, CA, USA) and an additional 20 min at 37°C incubation. The reactions were subjected to agarose gel electrophoresis and stained with ethidium bromide.

### scaRNA 9 3′ extension processing assays followed by qRT-PCR

ScaRNA 9 3′ extension transcript (100 nM) was incubated with GST (300 ng) or coilin (300 ng) in triplicate for 30 min at 37°C. The RNA/protein mix was then treated as above. Equal volumes of the reactions (0.5 µl) were also used for qRT-PCR analysis. The following primers were used to amplify specific regions of scaRNA 9 (labeled in Fig. 5B) (amplicon A) 5′-CTACAGTTGACCTGAGCTCA-3′ (forward) 5′-ACAGTTGCTGAAGATAATGG-3′ (reverse); (amplicon C) 5′-TAGCCAAATCTGAGCATCAGAAG-3′ (forward), 5′-TAAAACCTTTTCATCATTG-3′ (reverse), and (amplicon B) 5′-TAGCCAAATCTGAGCATCAGAAG-3′ (forward), 5′-ATAGTTTTGCTTCTCAGAACT-3′ (reverse). Brilliant II SYBR Green qRT-PCR master mix (Agilent, Santa Clara, CA, USA) was used for RNA analysis. The Stratagene Mx300P Real-Time PCR was used for RNA analysis and Microsoft Excel for post-hoc statistical analysis using the Student's T-test.

### Western blotting and antibodies

Western blotting was performed as described previously (Sun et al., 2005). The following antibodies were used for Western blotting: rabbit polyclonal α-coilin H300 (1:500) (Santa Cruz Biotechnology, Santa Cruz, CA), mouse monoclonal α-beta tubulin (1:1000) (Sigma–Aldrich, St Louis, MO), rabbit polyclonal α-WRAP53 antibodies (1:1000) (Bethyl laboratories, Montgomery, TX), mouse monoclonal anti-SMN (1:1000) (BD Transduction Laboratories, San Jose, CA) and mouse monoclonal α-GST (1:1000 Western) (Santa Cruz Biotechnology, Santa Cruz, CA).

### GST pull-down assays and RNAse A/T1 treatment

His-T7 tagged WRAP53, GST-tagged coilin and GST-tagged coilin fragments were partially purified from bacteria as described previously (Broome and Hebert, 2012; Broome and Hebert, 2013). GST only or GST-tagged proteins and His-WRAP53 were incubated for *in vitro* binding assay as described previously (Toyota et al., 2010) with few modifications. Briefly, the GST or GST-tagged proteins were untreated or treated with RNase A/T1 at 37°C for 30 min. The untreated or treated GST-proteins were then incubated with His-WRAP53 for 1 hour at 4°C with rocking. The beads were washed and subjected to electrophoresis, Western blotting and probing with anti-WRAP53 or anti-GST antibodies.

### RNA IPs, qRT-PCR and qPCR

For RNA IP, HeLa cells were transfected with control, WRAP53 or coilin siRNA. 48 h post transfection, cells were harvested and RNA IPs were set up as described previously (Broome and Hebert, 2013) using IgG (5 µg), α-coilin (5 µg) or α-WRAP53 (3 µg) antibodies. Equal volumes of the isolated RNA were used for the qRT-PCR analysis (Fig. 2). Primers for GAPDH, 47/45 S pre-rRNA, U2 snRNA and hTERC/hTR were previously reported (Broome et al., 2013; Broome and Hebert, 2013). Brilliant II SYBR Green qRT-PCR master mix (Agilent, Santa Clara, CA) was used for RNA analysis. The Stratagene Mx300P Real-Time PCR was used for RNA analysis and Microsoft Excel for post-hoc statistical analysis using the Student's T-test.

For the analysis of the scaRNAs associated with coilin or WRAP53 after knockdown, HeLa cells were transfected with control, WRAP53 or coilin siRNA. 48 h post transfection, cells were harvested and RNA IPs were set up as described previously (Broome and Hebert, 2013) using IgG (3 µg), α-coilin (3 µg) or α-WRAP53 (3 µg) antibodies. RNA isolated from the IPs was converted to cDNA using iScript cDNA kit (Bio-RAD, Hercules, CA, USA). Equal volumes of the cDNA were used for the qPCR analysis. The following primers were used to amplify specific regions of scaRNA2 (labeled in Fig. 3, amplicon A) 5′-GCGGAGCTGTGGCGTCGCGTGTGAGGC-3′ (forward), 5′-ACGATCCGATCAAATAAGATCAAAGTG-3′ (reverse), and scaRNA9 (labeled in Fig. 3, amplicon B) 5′-TAGCCAAATCTGAGCATCAGAAG-3′ (forward), 5′-TAAAACCTTTTCATCATTG-3′ (reverse), and (labeled in Fig. 3, amplicon C) 5′-TAGCCAAATCTGAGCATCAGAAG-3′ (forward), 5′-ATAGTTTTGCTTCTCAGAACT-3′ (reverse). Brilliant II SYBR Green qPCR master mix (Agilent, Santa Clara, CA) was used for RNA analysis. The Stratagene Mx300P Real-Time PCR was used for RNA analysis and Microsoft Excel for post-hoc statistical analysis using the Student's T-test.

### Telomerase activity assay and direct assessment of telomerase activity on IP beads

MEF26 and MEF42 cells were transfected with GFP vector only or GFP-mouse coilin (Fig. 6). 24 h post transfection, cells were lysed in 1× CHAPS buffer supplied by TRAPeze Telomerase Detection kit (Millipore, Billerica, MA, USA). Telomerase activities were measured according to the TRAPeze suggested protocol. Samples were resolved on a 12.5% polyacrylamide gel and stained with ethidium bromide. Densitometry of TRAP products was measured using Quantity One software. (Fig. 7) HeLa cells were transfected with control, WRAP53 or coilin siRNA. 24 h post transfection, cells were lysed in RIPA buffer (50 mM Tris-HCl, pH 7.6, 150 mM NaCl, 1% NP-40, 0.25% sodium deoxycholate, 0.1% SDS, and 1 mM ethylenediaminetetraacetic acid) containing protease inhibitor cocktail (Roche, Indianapolis, IN, USA). Cell lysates were briefly sonicated using the Fisher Scientific Sonic Dismembrator Model 100 and centrifuged at 16,000 g for 5 min at 4°C. The lysates were then immunoprecipitated with 5 µg of control IgG (control), 3 µg α-WRAP53 or 5 µg α-coilin antibodies. Complexes were captured by the addition of 50 µL of 50% Protein G Sepharose beads and rocking at 4°C for 18 h. The IP beads were washed 3 times with RIPA buffer and one time with 1× CHAPS buffer supplied by TRAPeze Telomerase Detection kit (Millipore, Billerica, MA, USA). Telomerase activities were measured according to the TRAPeze suggested protocol. Samples were resolved on a 12.5% polyacrylamide gel and stained with ethidium bromide, followed by quantification of TRAP products as described above.

## Supplementary Material

Supplementary Material
