# Supplementary material for: Coilin association with Box C/D scaRNA suggests a direct role for the Cajal body marker protein in scaRNP biogenesis
Source: Biol Open. 2014 Mar 21;3(4):240–9. doi: 10.1242/bio.20147443 (PMC3988793; doi:10.1242/bio.20147443)
Supplement: Supplementary Material [file supp_3_4_240__index.html]

Coilin association with Box C/D scaRNA suggests a direct role for the Cajal body marker protein in scaRNP biogenesis — Supplementary Material 

# Coilin association with Box C/D scaRNA suggests a direct role for the Cajal body marker protein in scaRNP biogenesis

## bio.20147443 Supplementary Material

**Files in this Data Supplement:**

- Supplementary Material - Isioma I. Enwerem et al. doi: 10.1242/bio.20147443
- Table S1 - Untreated gene summary
- Table S2 - Etoposide gene summary
- Table S3 - Actinomycin D gene summary
- Table S4 - Nocodazole gene summary
