# Supplementary material for: Coilin association with Box C/D scaRNA suggests a direct role for the Cajal body marker protein in scaRNP biogenesis
Source: Biol Open. 2014 Mar 21;3(4):240–9. doi: 10.1242/bio.20147443 (PMC3988793; doi:10.1242/bio.20147443)
Supplement: Supplementary Material [file supp_bio.20147443_bio.20147443-s1.pdf]

Biology Open (2014) 000, 1–10 doi:10.1242/bio.20147443

Supplementary Material
Isioma I. Enwerem et al. doi: 10.1242/bio.20147443

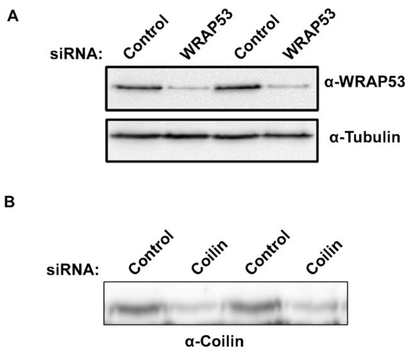

Fig. S1. (A) Cell lysates from knockdowns in Fig. 2A were subjected to SDS-PAGE and Western blot analysis, and the blot probed with α-WRAP53 or α-tubulin antibodies. (B) Cell lysates from the experiment in Fig. 2B were subjected to SDS-PAGE and Western blot analysis, and blot probed with α-coilin antibody.

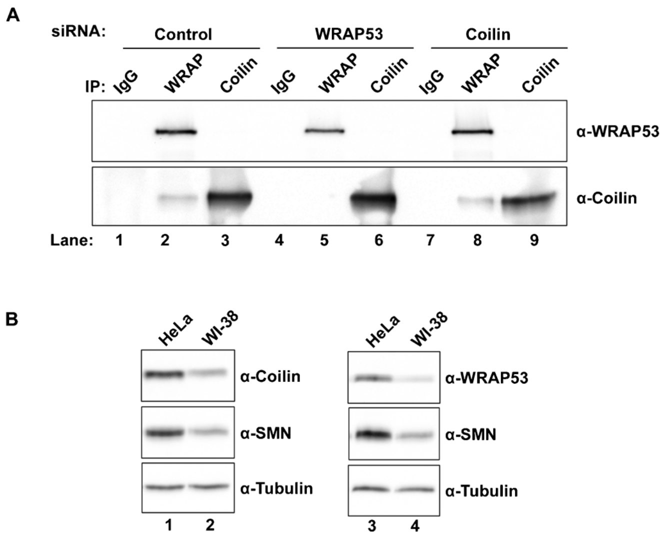

Fig. S2. (A) The IP beads used in Fig. 7 were subjected to SDS-PAGE and Western blot analysis, and the blots probed with α-WRAP53 or α-coilin antibodies. (B) Comparison of coilin, WRAP53 and SMN levels in HeLa and WI-38 cells. Equal amounts of total protein from HeLa and WI-38 cells were subjected to SDS-PAGE and Western blot analysis with the indicated antibody. Blots were probed with anti-tubulin as a loading control.
